# Supplementary material for: Resolving the full spectrum of human genome variation using Linked-Reads
Source: Genome Res. 2019 Apr;29(4):635–45. doi: 10.1101/gr.234443.118 (PMC6442396; doi:10.1101/gr.234443.118)
Supplement: Supplemental Material [file supp_gr.234443.118_Supplemental_longranger-2.2.zip › longranger-2.2.tar.bz2!/lr2.2-public/lib/stan/cmdstan/stan/doc/api/html/agrad_2rev_2matrix_8hpp.html]

Stan: src/stan/agrad/rev/matrix.hpp File Reference


|  |  |
| --- | --- |
|  | Stan  2.5.0  probability, sampling & optimization |


- Main Page
- Related Pages
- Namespaces
- Classes
- Files

- File List
- File Members

All Classes Namespaces Files Functions Variables Typedefs Enumerations Enumerator Friends Macros Pages

- src
- stan
- agrad
- rev

matrix.hpp File Reference

`#include <stan/agrad/rev/matrix/Eigen_NumTraits.hpp>`  
`#include <stan/agrad/rev/matrix/LDLT_alloc.hpp>`  
`#include <stan/agrad/rev/matrix/LDLT_factor.hpp>`  
`#include <stan/agrad/rev/matrix/crossprod.hpp>`  
`#include <stan/agrad/rev/matrix/determinant.hpp>`  
`#include <stan/agrad/rev/matrix/divide.hpp>`  
`#include <stan/agrad/rev/matrix/dot_product.hpp>`  
`#include <stan/agrad/rev/matrix/dot_self.hpp>`  
`#include <stan/agrad/rev/matrix/grad.hpp>`  
`#include <stan/agrad/rev/matrix/initialize_variable.hpp>`  
`#include <stan/agrad/rev/matrix/log_determinant.hpp>`  
`#include <stan/agrad/rev/matrix/log_determinant_ldlt.hpp>`  
`#include <stan/agrad/rev/matrix/log_determinant_spd.hpp>`  
`#include <stan/agrad/rev/matrix/log_softmax.hpp>`  
`#include <stan/agrad/rev/matrix/log_sum_exp.hpp>`  
`#include <stan/agrad/rev/matrix/mdivide_left.hpp>`  
`#include <stan/agrad/rev/matrix/mdivide_left_ldlt.hpp>`  
`#include <stan/agrad/rev/matrix/mdivide_left_spd.hpp>`  
`#include <stan/agrad/rev/matrix/mdivide_left_tri.hpp>`  
`#include <stan/agrad/rev/matrix/multiply.hpp>`  
`#include <stan/agrad/rev/matrix/multiply_lower_tri_self_transpose.hpp>`  
`#include <stan/agrad/rev/matrix/quad_form.hpp>`  
`#include <stan/agrad/rev/matrix/sd.hpp>`  
`#include <stan/agrad/rev/matrix/softmax.hpp>`  
`#include <stan/agrad/rev/matrix/sort.hpp>`  
`#include <stan/agrad/rev/matrix/squared_distance.hpp>`  
`#include <stan/agrad/rev/matrix/stan_print.hpp>`  
`#include <stan/agrad/rev/matrix/stored_gradient_vari.hpp>`  
`#include <stan/agrad/rev/matrix/sum.hpp>`  
`#include <stan/agrad/rev/matrix/tcrossprod.hpp>`  
`#include <stan/agrad/rev/matrix/to_var.hpp>`  
`#include <stan/agrad/rev/matrix/trace_gen_inv_quad_form_ldlt.hpp>`  
`#include <stan/agrad/rev/matrix/trace_gen_quad_form.hpp>`  
`#include <stan/agrad/rev/matrix/trace_inv_quad_form_ldlt.hpp>`  
`#include <stan/agrad/rev/matrix/trace_quad_form.hpp>`  
`#include <stan/agrad/rev/matrix/typedefs.hpp>`  
`#include <stan/agrad/rev/matrix/value_of.hpp>`  
`#include <stan/agrad/rev/matrix/variance.hpp>`

Go to the source code of this file.


---

[ Stan Home Page ]

*© 2011–2014,
Stan Development Team.*
